# An Intact Cell Bioluminescence-Based Assay for the Simple and Rapid Diagnosis of Urinary Tract Infection

**DOI:** 10.3390/ijms21145015

**Published:** 2020-07-16

**Authors:** Sherwin Reyes, Nga Le, Mary Denneth Fuentes, Jonathan Upegui, Emre Dikici, David Broyles, Edward Quinto, Sylvia Daunert, Sapna K. Deo

**Affiliations:** 1 Department of Biochemistry and Molecular Biology, University of Miami Miller School of Medicine, Miami, FL 33136, USA; sxr1145@miami.edu (S.R.); ntl21@miami.edu (N.L.); jupegui@miami.edu (J.U.); EDikici@med.miami.edu (E.D.); DBroyles@med.miami.edu (D.B.); SDaunert@med.miami.edu (S.D.); 2 The Dr. John T. McDonald Foundation Bionanotechnology Institute of University of Miami, Miami, FL 33136, USA; 3 FEU-Nicanor Reyes Medical Foundation, Institute of Medicine, West Fairview, Quezon City 1118, Philippines; marydenfuentes@gmail.com; 4 The Graduate School, University of Santo Tomas, España Manila 1015, Philippines; edwardquinto@gmail.com; 5 Clinical and Translational Science Institute of University of Miami, Miami, FL 33136, USA

**Keywords:** urinary tract infection diagnosis, bioluminescence, biosensor, rapid detection

## Abstract

Urinary tract infection (UTI) is one of the most common infections, accounting for a substantial portion of outpatient hospital and clinic visits. Standard diagnosis of UTI by culture and sensitivity can take at least 48 h, and improper diagnosis can lead to an increase in antibiotic resistance following therapy. To address these shortcomings, rapid bioluminescence assays were developed and evaluated for the detection of UTI using intact, viable cells of *Photobacterium mandapamensis* USTCMS 1132 or previously lyophilized cells of *Photobacterium leiognathi* ATCC 33981™. Two platform technologies—tube bioluminescence extinction technology urine (TuBETUr) and cellphone-based UTI bioluminescence extinction technology (CUBET)—were developed and standardized using artificial urine to detect four commonly isolated UTI pathogens—namely, *Escherichia coli*, *Proteus mirabilis*, *Staphylococcus aureus*, and *Candida albicans*. Besides detection, these assays could also provide information regarding pathogen concentration/level, helping guide treatment decisions. These technologies were able to detect microbes associated with UTI at less than 10^5^ CFU/mL, which is usually the lower cut-off limit for a positive UTI diagnosis. Among the 29 positive UTI samples yielding 10^5^–10^6^ CFU/mL pathogen concentrations, a total of 29 urine specimens were correctly detected by TuBETUr as UTI-positive based on an 1119 s detection window. Similarly, the rapid CUBET method was able to discriminate UTIs from normal samples with high confidence (*p* ≤ 0.0001), using single-pot conditions and cell phone-based monitoring. These technologies could potentially address the need for point-of-care UTI detection while reducing the possibility of antibiotic resistance associated with misdiagnosed cases of urinary tract infections, especially in low-resource environments.

## 1. Introduction

Urinary tract infection (UTI) is a common bacterial infection of the urethra, bladder, ureters, and the kidneys, affecting approximately 150 million people annually worldwide [1,2,3]. A significant cause of UTI is any pathogen found in the urine with a bacterial load of greater than 100,000 CFU/mL. *Escherichia coli (E.coli)* is the most common pathogen that infects the urinary tract, causing 90% of community-acquired UTI and 50% of hospital-acquired UTI. The diagnosis of UTI typically takes at least two days, even with the availability of modern technologies [4,5]. Recurrent UTI poses a particular threat to patients with acute UTI episodes [1,3], while misdiagnosed infection can seriously increase the prevalence of antimicrobial resistance. Therefore, a rapid and cost-efficient diagnostic test to identify UTI is important, especially for low-income countries, wherein access to expensive technologies is difficult, and on-site testing is preferable. Several modern technologies have been tested for application in UTI diagnosis. One approach is using biosensors for rapid point-of-care testing [6]. A biosensor is a device that translates biological recognition of a specific pathogen into a measurable signal [6]. Biosensors (electrochemical, optical, enzymatic, and microbial) show high efficacy in this application by combining pathogen identification and testing for antimicrobial susceptibility. Results can be seen from 1 to 3.5 h, but biosensors have the drawback of a fairly significant cost when compared against conventional methods. Urinalysis results, particularly the leukocyte esterase (LE), [7,8,9] nitrite test (NT), and some other point-of-care platforms, such as uricult trio, uriscreen, and urisys 1100 [9,10], are often utilized to determine the severity of the UTI infection as well as to diagnose probable cystitis [10]. However, urinalysis may not give a correct impression of the infection due to factors, such as contamination with various normal flora of the genitourinary tract and misinterpretation of the results [9,11,12,13]. In addition, although uricult trio, uriscreen, and urisys 1100 are reliable point-of-care testing devices, they are expensive and/or require additional equipment [10]. Other UTI detection technologies include initial screening assays, such as lateral flow immunoassay and flow cytometry. However, lateral flow assays often suffer from sensitivity issues, and flow cytometry is notoriously time-consuming. Fluorescence in situ hybridization (FISH) assays and microfluidic devices can provide rapid UTI detection but cannot currently be used in point-of-care settings [14]. Quorum sensing-based detection technologies could potentially be used in detecting bacterial infections, such as UTI, since they have been developed to monitor bacterial infections in gastrointestinal (GI) diseases, such as Crohn’s and ulcerative colitis [15,16]. As such, the need for rapid UTI detection with amenable on-site testing is a necessity [14]. We approached this need through the design and development of simple bioluminescence-based assays that required only living, unicellular bioluminescent organisms that could be kept for the long term in a dry form for the diagnosis of UTI.

Whole-cell bioluminescence is easy to monitor; it does not need an excitation source. This method can be adapted to point-of-care set-up and is easier to use, while the sensitivity of bioluminescent detection is better compared to other optical methods owing to the high signal to noise ratio. As opposed to fluorescence, the lack of optical excitation yields superior detection limits due to the near absence of background. Bioluminescent bacteria are prokaryotes that can produce their own light solely from their unique metabolic activity. Marine luminous bacteria have been studied and extensively utilized in the biotechnological detection of toxic chemicals, pollutants, pesticides, dissolved oxygen, freshness, quorum sensing, ATP, and other physicochemical conditions [17,18,19,20,21,22,23]. Bioluminescence is living light; the partial or complete inhibition depends on the cells’ viability, which is affected by the cells’ immediate physicochemical condition and/or direct exposure to toxicants. Thus, bioluminescence lends itself well as a biological sensing tool for the direct observation and measurement of cell viability and well-being [18,19,24]. In addition, it contributes to important applications in other scientific disciplines, particularly in the field of diagnostic medicine. For our studies, two bioluminescent bacteria, *Photobacterium mandapamensis* USTCMS 1132 (*P. mandapamensis*) and *Photobacterium leiognathi* ATCC 33981^TM^ (*P. leiognathi*)—isolated from a luminescent squid collected in the Philippines and acquired from American Type Culture Collection (ATCC), respectively—were used to develop bioluminescence-based assays that allow direct diagnosis of UTIs [25,26].

Using an intact, viable luminescent cell suspension could provide a simpler, more rapid, and inexpensive method for the detection of urinary tract infection. Based on our findings that exposure to infected urine resulted in the loss of organismal bioluminescence, we developed a bioluminescence-based cell suspension assay that could be clinically validated against currently accepted, conventional assays for UTI, such as the culture technique. With the use of standardized suspensions of luminous bacteria, infections in the urinary tract could be rapidly detected in less than 2 h compared to conventional urine culture that can take up to two days. Our whole-cell bioluminescence assay was adapted to two different platforms, a laboratory equipment-based detection called tube bioluminescence extinction technology urine (TuBETUr) and a point-of-care, cellphone-based detection termed cellphone-based UTI bioluminescence extinction technology (CUBET). The TuBETUr and CUBET assays worked by simply measuring the luminescence of *P. mandapamensis* or *P. leiognathi*, respectively. The loss of brightness indicated the detection of a bacterial UTI; conversely, a constant brightness measured beyond a certain cut-off time indicated a negative UTI. Both gave rapid results using the bioluminescent organism as the only reagent, making TuBETUr and CUBET extremely inexpensive and effective UTI diagnostic tools.

## 2. Results and Discussion

### 2.1. Identification of Bioluminescent Bacteria

Following isolation of *Photobacteria* from bigfin reef squid (*Sepioteuthis lessoniana*), the phenotypic characteristics of individual colonies of the plump rod-shaped bacteria were assessed, as shown in Table S1. For confirmation, colonies were picked and expanded for lysis and recovery of total RNA. Genotypic characterization was then performed by Macrogen (Seoul, South Korea) using 16S rRNA sequencing on four of the brightest samples of luminous bacteria (Table S2). A BLAST search of the almost complete 16S rRNA gene sequence (1472 bp) from isolate 1 was performed against data from the National Center for Biotechnology Information (http://www.ncbi.nlm.nih.gov), and the sequence with the highest homology was retrieved from GenBank. Based on the partial rRNA sequence, an alignment of 99% was attributed to *P. mandapamensis* (ATCC 33981) with the accession number: AY341442 (max score = 1718, query coverage = 100%, *E* value = 0, max identity = 99%). The isolate was deposited to the University of Santo Tomas culture collection with the assigned accession number of USTCMS 1132 (*P. mandapamensis*).

### 2.2. Tube Bioluminescence Extinction Technology for Urine (TuBETUr)

Tube bioluminescence extinction technology for urine (TuBETUr) was designed as a test tube assay that used a whole-cell, bioluminescence-based bacterial sensor to detect other bacteria in the urine. In TuBETUr, the bioluminescent cells underwent complete light inhibition (or blackout) when mixed with other bacterial cells, especially highly metabolic uropathogenic bacteria. To quantify the uropathogen, a fixed suspension of bioluminescent cells was mixed with a viable cell suspension of uropathogen in a test tube, and the time of complete blackout was measured. The addition of active bioluminescent *P. mandapamensis* cells into an active culture of other bacteria reproducibly led to this phenomenon. In addition, an indirect correlation between UTI pathogen cell density and time-to-blackout was observed, where increasing pathogen density decreased the overall assay time. Therefore, TuBETUr could be harnessed to generate a dose-response curve and enabled quantification of the pathogen cell density, as previously described, using an ATP-bioluminescence assay under similar conditions [20,21,27,28,29]. The TuBET method was first described by Quinto (2007) to quantify spoilage bacteria in fish and from milk samples [30,31].

By extending the matrix capabilities of TuBET to crude urine, the detection of pathogenic bacterial load in artificial urine could be realized at concentrations ≥ 10^5^ CFU/mL. For this purpose, several luminous marine bacterial species were isolated from squid and screened for bright and stable bioluminescence. One strain exemplifying these characteristics was chosen and further characterized as the potential biological reagent for the TuBETUr assay. The isolate was identified as *P. mandapamensis* based on partial phenotypic characterization and almost complete rRNA gene sequencing technique.

### 2.3. Relationship between the Cell Density of Selected Uropathogens and Time-To-Blackout in Artificial Urine

**Figure 1:**
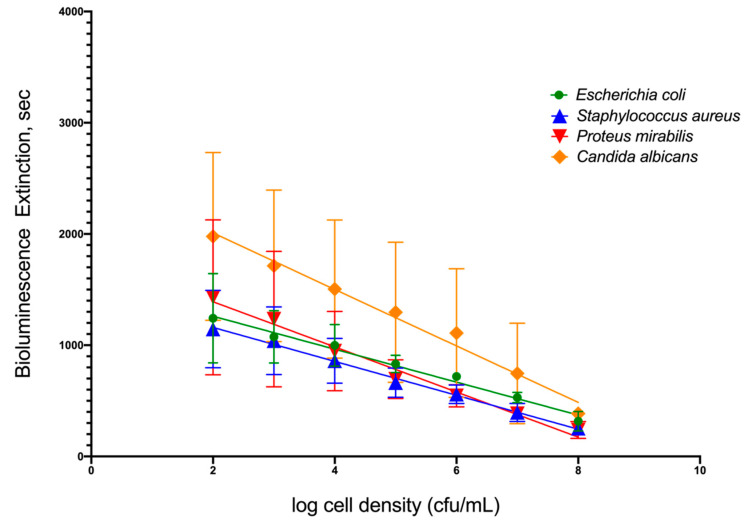
Relationship of blackout time and approximate log_10_ cell density/mL of the four common uropathogens, which included *Escherichia coli* ATCC 25922^TM^, *Staphylococcus aureus* ATCC 23235^TM^, *Proteus mirabilis* ATCC 35659^TM^, and *Candida albicans* ATCC 14053^TM^ in artificial urine.

To simulate infection in patients’ urine, four common uropathogenic bacteria strains were utilized as standard organisms to determine the capability of TuBETUr technology in detecting bacteria spiked within a range of 10^2^–10^8^ CFU/mL in artificial urine. Each uropathogen showed a linear decrease in light intensity as the number of cells present in the artificial urine increased (Figure 1). Clinically, a 10^5^ CFU/mL concentration [32,33,34], although indicating a significant number of uropathogens, is considered the lower limit of detection. Any bacterial concentration in the urine at or above this limit is considered serious enough to warrant treatment, whereas any bacterial cell count below this limit is considered negative. Thus, the lower limit of detection (Table 1) was determined by assigning a value, termed the cut-off time, that statistically defines an assay detection limit, as determined by the longest pathogen blackout time. The cut-off time, therefore, represents a point that is three standard deviations beyond the longest experimental mean blackout time for a culture containing ≥ 10^5^ CFU/mL bacteria and defines an arbitrary “cut-off”, beyond which all samples are considered negative. Among the four uropathogens, *Candida albicans*, an opportunistic fungus associated with moniliasis in the urinary tract, showed the longest blackout time in this assay. Summarizing the data found in Table 1 and shown graphically in Figure S1, the mean blackout times for 10^5^ CFU/mL of *E. coli*, *S. aureus*, *P. mirabilis,* and *C. albicans* uropathogens in artificial urine were 831, 663, 694, and 1246 s, respectively. As a result, an assay cut-off time of 1257 s was utilized for TuBETUr in the diagnosis of UTI, as this mean blackout time represents ≥ 10^5^ CFU/mL concentration for any of the uropathogenic cell lines tested in the urine.

### 2.4. Application of TuBETUr for UTI Diagnosis

**Figure 2:**
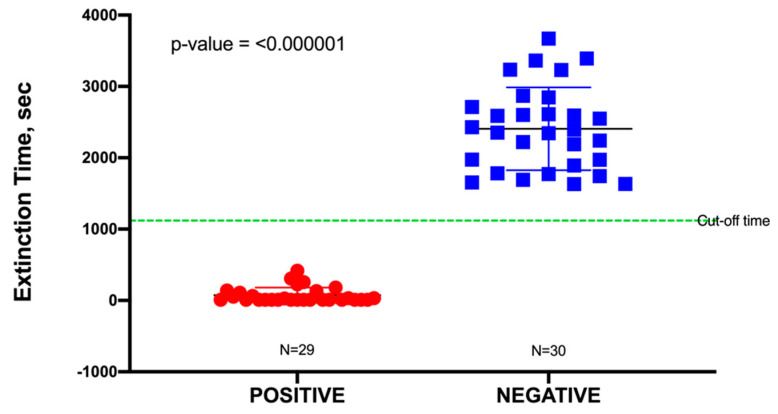
Sample distribution of urinary tract infection (UTI) negative and positive samples detected by tube bioluminescence extinction technology urine (TuBETUr) technology compared to standard urine culture.

In this study, a total of 60 urine specimens were used, comprising 30 negative samples and 30 positive samples representing uncomplicated UTIs, although one outlier in the positive data was identified from a Dixon’s *Q* test and was subsequently removed. Uncomplicated urine specimens were used to minimize false positives due to potential interference (toxicity) from any unknown pharmaceuticals present. The pH of these urine samples was found to range from 6.0 to 7.5; this had no significant effect on the blackout times, as most bacteria would readily grow in a pH range of 6.0–8.0. As shown in Table S3, all positive urine specimens yielded a culture of ≥10^5^ CFU/mL, which was an indication of a clinically positive UTI. Blackout times ranged from as few as 10 to 1110 s, and the 29 urine specimens were correctly identified by TuBETUr (Table S3). As the blackout times for the 29 validated positive samples fell below the cut-off time of 1257 s expected for 10^5^ CFU/mL, the actual concentrations were likely much higher (Table 1). The blackout times for negative specimens were all above the arbitrary 1257 s cut-off time for the ≥ 10^5^ log_10_ CFU/mL detection limit and were correctly diagnosed by TuBETUr with a specificity of 100%. Thus, TuBETUr provided a sensitivity for positive UTI diagnosis of 29/29 or 100% (Figure 2). These results confirmed TuBETUr to be an exceptional diagnostic method for UTI. Further, the substantially longer blackout times for negative samples indicated that the upper time limit could potentially be adjusted to 1257 s in order to prevent further false negatives. Although TuBETUr was not designed to identify the species of microorganisms present, this was not a drawback as quantification of cell density in the urine is still considered the most important parameter in diagnosing UTI.

As seen in Table S3, 14 (46.67%) of the positive UTI samples were caused by *E. coli*, which was anticipated due to this being the most common species identified in UTIs. In this study, 3 of the 29 positive samples (10.34%) were found to contain less common non-lactose and/or non-fermentative bacteria, such as *Acinetobacter baumannii*, *Burkholderia cepacia*, and *P. mirabilis*. Aside from those Gram-negative pathogens, Gram-positive organisms like *Staphylococcus aureus* and *Enterococcus faecalis* were also identified and accounted for 6.90% of the population. Although it is also known that the predominant infective microorganism determines the rate and severity of the infection, mixed cultures can complicate recovery and treatment options. However, only 2 out of the 29 UTI-positive samples (6.90%) screened as mixed cultures and were shown to contain *E. coli* with either *Citrobacter freundii* or *Acinetobacter baumannii*.

### 2.5. Optimization of Lyophilization Conditions for Photobacterium leiognathi ATCC 33981^TM^

**Figure 3:**
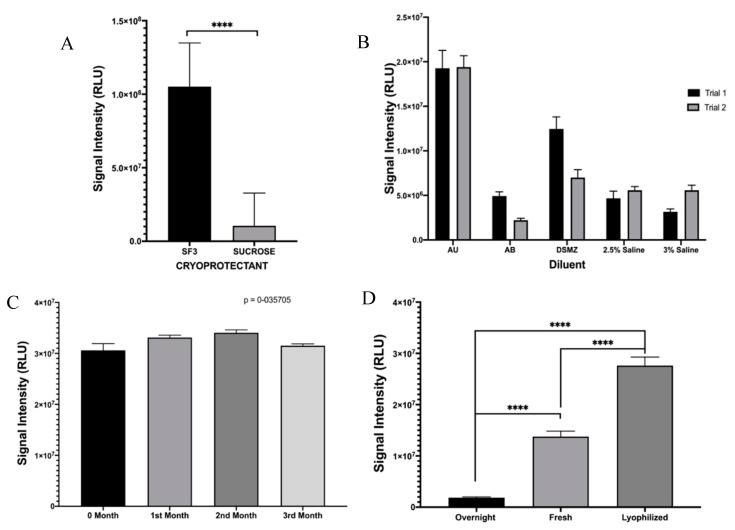
Optimization of lyophilized cells of *P. leiognathi*. Bioluminescence activity was assessed following (**A**) exposure to cryoprotectant (**** *p* ≤ 0.0001) or (**B**) varying diluent composition. (**C**) Bioluminescence of lyophilized cells using SF3 cryoprotectant was determined over 3 months, while (**D**) the normalized signal from overnight/fresh/lyophilized cultures of *P. leiognathi* was compared (**** *p* ≤ 0.0001).

In order to transition the TuBETUr assay to a point-of-care platform, it was necessary to consider lyophilization as a means of preserving the whole-cell bioluminescent reporter until use. As *P. mandapamensis* USTCMS 1132 has not yet been commercialized at the time of publication, we decided to incorporate the phenotypically identical and readily available *P. leiognathi* (ATCC 33981^TM^) [25,26,35,36] as a comparable substitute, which would be ideal for direct adaption of this technology for UTI diagnosis. Two suitable cryoprotectants (SF3 is composed of (120 g/L lactose, 20 g/L soluble starch, and 10 g/L sodium chloride dissolved in deionized water and adjusted to pH 7) and sucrose) [37] were tested against high concentrations (~OD_600_ = 1.5) of *P. leiognathi* for lyophilization, and SF3 was found to provide significantly higher retention of bioluminescence as compared to sucrose (Figure 3A). Likewise, various salinized buffers were compared, and the best bioluminescence values achieved for previously lyophilized *P. leiognathi* cells (~1.8–2.0 × 10^7^ RLU) were fortuitously achieved using artificial urine (Figure 3B). Lyophilized cells were also tested for bioluminescence stability and light intensity following reconstitution over 3 months, and values about 3.0 × 10^7^ RLU were obtained—indicating that the lyophilization conditions were well-optimized for *P. leiognathi,* allowing for long term storage of the bacteria at room temperature in a dry form (Figure 3C). Additionally, bioluminescence intensities from an overnight culture, fresh culture (OD_600_ = 0.711), and reconstituted *P. leiognathi* cells at identical concentrations were compared. It was observed that the bioluminescence intensity from the previously lyophilized cells was higher when compared to both overnight and fresh cultures. Thus, lyophilization was an efficient means of preserving cells and would not negatively impact the sensitivity of a point-of-care assay (Figure 3D).

### 2.6. Microtiter Plate Assay Using Lyophilized Photobacterium leiognathi

**Figure 4:**
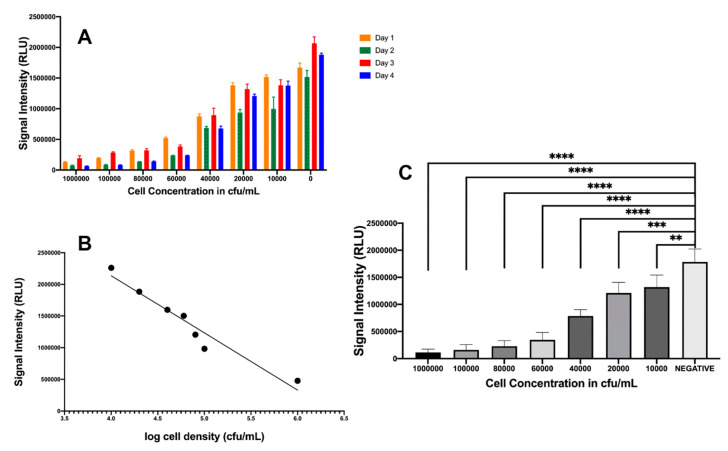
Relationship of signal intensity from *P. leiognathi* with various cell concentrations of *E. coli*. (**A**) UTI assay for 4 consecutive days. (**B**) Bioluminescence assay for detecting *E. coli* at various concentrations. A linear relationship between log cell density and bioluminescent signal. (**C**) ANOVA of UTI assay. A decrease in luminescence was measured after 5 min incubation at room temperature. ** *p* = 0.0023, *** *p* = 0.0002, **** *p* ≤ 0.0001. Statistical data analysis was performed using GraphPad Prism version 8.3.4 for Mac OS, GraphPad Software, San Diego, CA, USA.

Verification of similar blackout characteristics for *P. leiognathi* was performed by spiking uropathogenic *E. coli* in artificial urine and reconstituting lyophilized *P. leiognathi* cultures. Signal intensities were monitored with a luminometer (CLARIOstar, BMG Labtech) and were found to decrease in a dose-dependent manner, as shown in Figure 4A–C. These plots showed a significant difference (*p* < 0.0001) in light intensity compared to the negative control in the presence of *E. coli* cell concentrations ranging from 10^6^–10^3^ CFU/mL, based on Prism analysis using ANOVA, followed by Dunnett’s test for multiple comparisons. Figure 4B demonstrates the inverse correlation between signal intensity and cell density and provides an acceptable R^2^ value of 0.93 (*y* = 9.0 × 10^5^*x* + 5.0 × 10^6^). This further indicated that *P. leiognathi* provided an excellent replacement for *P. mandapamensis* and enabled its use in the development of the CUBET technology for simple and efficient point-of-care UTI detection.

### 2.7. Mechanism of Action for Bioluminescence Inhibition

Most bioluminescence associated with marine bacteria is based on quorum sensing. Quorum sensing is a way that microbes communicate in order to time the production of important chemicals and enzymes necessary for their survival; this also includes chemicals and enzymes responsible for infectivity and pathogenicity on Gram-positive and Gram-negative bacteria [38,39]. Uropathogens are capable of producing toxins that can alter and affect normal bodily functions, and this toxin production is essential for their infectivity and pathogenicity. However, the production of bioluminescence in the genus *Photobacteria* is not associated with quorum sensing [23,40]. Further, data gathered on the effects of high concentrations of non-pathogenic *E. coli* cells on *P. leiognathi* signal intensity demonstrated that neither quorum sensing nor toxin production was responsible for the blackout phenomenon seen with uropathogens. At high concentrations of NEB^®^ 5-alpha, *P. leiognathi* bioluminescence was shown to decrease (Figure S2A), but NEB^®^ 5-alpha cells did not produce two important quorum sensing molecules—acyl homoserine lactone (AHL) and Autoinducer-2 (AI2)—and were not pathogenic. Given that NEB^®^ 5-alpha could initiate blackout, albeit, with a higher concentration, it was deduced that oxygen depletion must be the mitigating factor during a blackout. Bioluminescence production requires oxygen, and the presence of any pathogenic organism in urine that consumes oxygen will result in the inhibition/blackout of bioluminescence. This was confirmed by repeating the standard blackout test in deoxygenated urine, where a significant drop in signal intensity was observed for oxygen-starved *P. leiognathi* when compared to their oxygenated counterparts (Figure S2B,C). Bacteria infecting the urinary tract are primarily aerobic and facultative anaerobic microorganisms and would rely heavily on oxygen for their proliferation [41,42]. With a demonstration that oxygen depletion in the solution resulted in an essentially identical decrease in signal intensity, it was likely that natural deoxygenation as a result of metabolism was the primary mechanism behind the blackout phenomenon. As this was not found to correlate with pH changes, the blackout phenomenon should be unaffected by natural variations in urine, minimizing all non-physiological variables besides the bacterial load. Given that bacteria would only be found in urine during a urinary tract infection, an indirect method of identifying the presence of bacterial contamination was sufficiently robust for the diagnosis of UTI.

### 2.8. Cellphone-Based Urinary Tract Infection Bioluminescence Extinction Technology (CUBET)

**Figure 5:**
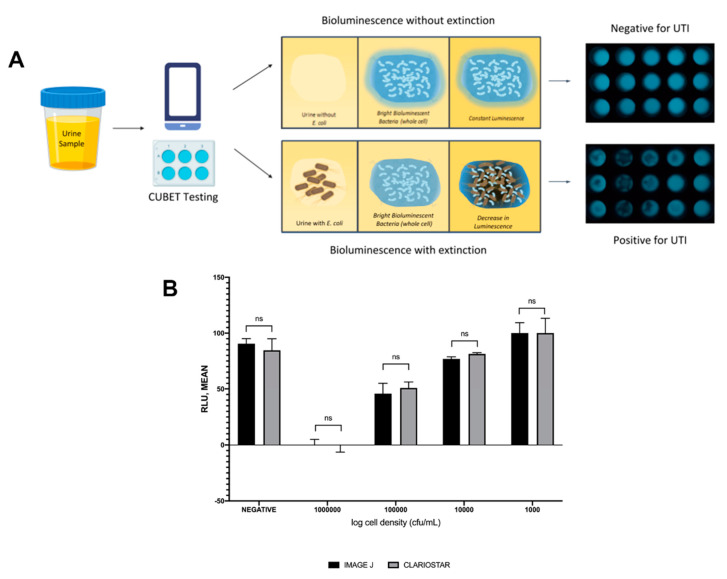
(**A**) Flow of urinary tract infection detection using cellphone-based UTI bioluminescence extinction technology (CUBET), (**B**) Comparison of the CUBET assay with a highly sensitive luminometer. (ns = not significant)

Upon demonstration that lyophilized *P. leiognathi* cells could be used as the bioluminescent signaling reagent for UTI detection, we developed a point-of-care device to aid in diagnosing urinary tract infections in remote areas or high-traffic clinics. The CUBET device was 3D printed based on a schematic rendered in Rhinoceros^®^ (v. 6) software. As shown in Figure 5A, the CUBET assay did not require sophisticated instrumentation beyond a simple camera-equipped cellphone to confirm suspected UTIs in patients. Furthermore, the results obtained from CUBET were comparable to that of our previous instrumental results with greater than 99% confidence. Figure 5A demonstrates the simplicity of the process, and the inclusion of lyophilized reporter enabled the long-term storage of the assay kit. Within minutes, CUBET could detect if a patient had a UTI by simply adding the urine sample to the chamber and waiting. If the *P. leiognathi* did not blackout after 5 min, then the sample was negative for UTI, and positive samples could be detected with high sensitivity (Figure 5B) within the first 5 min of adding a urine specimen.

## 3. Materials and Methods

### 3.1. Formulation of the Agar Medium for Bioluminescent Bacteria

Two different media (trypticase yeast extract seawater agar (TYESA) and AB medium) were used in this study. A suitable agar medium (TYESA medium) necessary to support the growth and production of bioluminescent light was prepared by using 30 g of tryptone, 20 g of Bacto agar and 5 g yeast (BD Biosciences; San Jose, CA, USA), per liter of deionized water. To selectively support the growth of the bioluminescent microorganism, artificial seawater was prepared by adding 30 g/L of analytical grade sodium chloride (EMD Chemicals; Gibbstown, NJ, USA). One liter of AB medium was prepared using (3 mL glycerol, 5 g casitone, 3 g yeast extract (BD Biosciences; San Jose, CA, USA), 1.179 g potassium phosphate monobasic (Sigma-Aldrich; St. Louis, MO, USA), 5.395 g sodium phosphate dibasic dihydrate (Honeywell Fluka; Muskegon, MI, USA, 30 g sodium chloride (EMD Chemicals; Gibbstown, NJ, USA), and 18 g Bacto agar (BD Biosciences; San Jose, CA, USA).

### 3.2. Isolation of Bioluminescent Bacteria

Approximately 1 kg of bigfin reef squid (*Sepioteuthis lessoniana*) was procured from a local market in Quezon City, Philippines. In the laboratory, an incision was made on the ventral portion of the squid’s mantle, exposing its long slender plastic-like pen. The pen was removed, and the head was pulled with its gastrointestinal (GI) tract [43]. A representative swab of the GI tract was streaked on TYESA plates using the multiple-interrupted method. For the incubation, the plate was wrapped with paper and stored at room temperature for 16–24 h. After incubation, the plated culture was observed in a dark room to check if there were bioluminescent colonies. If no bioluminescence was evident, the procedure was repeated using a fresh batch of squid. If positive for bioluminescence, the bright luminous colonies were isolated using a sterile dissecting needle and subsequently cultured on TYESA slants for reference and storage.

### 3.3. Identification of Bioluminescent Bacteria from Squid (Phenotypic)

Each of the bioluminescent isolates was inoculated into various media: salt tolerance assay (0 %NaCl, 3% NaCl, 8% NaCl, 10% NaCl (EMD Chemicals; Gibbstown, NJ, USA), (alkaline peptone water (APW), sulfide indole motility medium (SIM), trypticase soy broth (TSB) with glycerol, TSB with tyrosine, and thiosulfate citrate bile salts sucrose (TCBS) agar, glucose, lactose, maltose, and mannitol (BD Biosciences; San Jose, CA, USA) tube for biochemical identification. These media, after inoculation, were incubated at 37 °C for 16–24 h and observed based on characteristic colony growth and color change of the medium. Turbidity indicated growth on APW, salt tolerance assay, glycerol, and tyrosine. For the carbohydrate utilization assay (glucose, lactose, maltose, and mannitol), a change from red to the yellow color indicated a growth of acid-producing bacteria and was recorded as positive. Furthermore, results from TCBS were based on colonial growth and color change of the media. Growth in TCBS with a color change of the media from green to yellow was considered due to a sucrose-fermenting bacterium, while no color change represented non-sucrose-fermenting bacteria. Other media, such as skimmed milk (Oxoid, Thermo-Fisher; San Jose, CA, USA) with 2.8% NaCl (EMD Chemicals; Gibbstown, NJ, USA), were also attempted. Due to the original turbidity of the media after incubation, re-inoculation on nutrient agar and subsequent incubation were required for growth determination [44].

### 3.4. Identification of Bioluminescent Bacteria (Genotypic)

DNA extracted from four brightly luminous bacterial isolates were sent to Macrogen (Seoul, South Korea) for 16S rRNA sequencing.

### 3.5. Preparation of Luminous Bacterial Suspension in 2.5% (w/v) Saline

To prepare a suspension of the luminous bacteria, 10 mL of 2.5% saline was poured on a 15 h agar culture of *P. mandapamensis*, and the colonies were gently scraped loose. The suspension was then transferred to a 250 mL Erlenmeyer flask. A 10^10^ CFU/mL suspension was subsequently prepared and diluted with saline to prepare a 2 McFarland turbidity-adjusted cell suspension.

### 3.6. Urine Samples

Urine samples, representing 60 individuals, were obtained from Far Eastern University–Nicanor Reyes Medical Foundation Clinical Laboratory Bacteriology and Clinical Microscopy section, Quezon City Philippines. Samples were acquired as a gift following the laboratory assessment of biosafety and deidentification.

### 3.7. Preparation of Artificial Urine (AU-Siriraj)

Artificial urine was prepared based on the formulation of Siriraj [45] to closely mirror the physiological components and concentrations found in normal human urine. Artificial Urine-Siriraj (AU-Siriraj) was prepared by dissolving 2.427 g of urea, 0.034 g of uric acid, 0.090 g of creatinine, 0.297 g of trisodium citrate dihydrate, 0.634 g of sodium chloride, 0.450 g of potassium chloride (KCl), 0.161 g of ammonium chloride, 0.089 g of calcium chloride dihydrate (CaCl2·2H2O), 0.100 g of magnesium sulfate heptahydrate, 0.034 g of sodium bicarbonate, 0.003 g of sodium oxalate, 0.258 g of sodium sulfate, 0.100 g of sodium phosphate monobasic, and 0.011 g of sodium phosphate dibasic (all chemical reagents from Sigma-Aldrich; St. Louis, MO, USA) in 200 mL of deionized water [45].

### 3.8. Bioluminescence Assay Using Reference Organisms in Artificial Urine for the Tube Bioluminescence Extinction Assay Urine (TuBETUr)

Ten-fold serial dilutions ranging from 10^2^–10^8^ CFU/mL of *Escherichia coli* ATCC 25922^TM^, *Staphylococcus aureus* ATCC 23235^TM^, *Proteus mirabilis* ATCC 35659^TM^, and *Candida albicans* ATCC 14053^TM^ cultures were individually grown in artificial urine (AU-Siriraj) for several hours, salinized in 2.5% (*w*/*v*) sodium chloride (EMD Chemicals; Gibbstown, NJ, USA), and mixed with a 2 McFarland standard bioluminescent cell suspension of *P. mandapamensis*. In practice, this translated to 9 mL of each cell suspension prepared as above, mixed with a 1 mL aliquot of 10^10^ CFU/mL saline suspension of *P. mandapamensis*. The tube was capped and inverted once, and the time-to-blackout in min was recorded per dilution level for each culture. The result was graphed against the approximate cell density determined for the uropathogens and expressed as CFU/mL.

### 3.9. Tube Bioluminescence Extinction Technology for Urine (TuBETUr) Samples

The bioluminescence extinction (blackout time) for the 60 urine samples was examined. These samples were initially collected from patients with uncomplicated UTI. The urine samples were coded and measured for their pH and turbidity. The estimated viable plate count of each urine sample was determined side by side as the blackout time was being determined. Freshly collected urine samples were prepared identically to the artificial urine samples in Section 3.8. Blackout was observed visually in a dark room, and photographs were obtained. From the blackout period of the 59 remaining uncomplicated urine samples (following outlier removal), a cut-off time was determined from the 3-sigma rule applied to the longest positive blackout time that would distinguish normal (non-infected) urine from the UTI-diagnosed samples. Infected urine samples were those that yielded an estimated viable plate count of 10^5^ CFU/mL. Non-infected urine had an estimated viable plate count of less than 10^5^ CFU/mL. From this sample size, the mean time-to-blackout for the normal and UTI-infected urine samples was determined and compared statistically to the results obtained for the 10^5^ CFU/mL standards in artificial urine.

### 3.10. Assay to Determine the Mechanism of Action

#### Determination of Acyl Homoserine Lactone (AHL), Autoinducer 2 (AI2), and Bacterial Toxin Production Effects on Non-Pathogenic *E. coli*

Overnight cultures of *E. coli* NEB5-alpha competent cells (New England Biolabs; Ipswich, MA, USA) were serially diluted in 10-fold steps from 10^6^ to 10^3^ CFU/mL and spiked into artificial urine, which served as the test sample. In a sterile, black 96-well flat bottom (chimney well) Cellstar^®^TC plate, 10 μL of freshly reconstituted *P. leiognathi* cells and 190 μL of the incubated cells were added to triplicate wells for each dilution. The plate was then mixed and read by a luminometer.

### 3.11. Detection of UTI in Artificial Urine Using the CUBET Assay

The CUBET platform was prepared and set-up in the laboratory. The 200 μL of the negative control (DSMZ (Deutsche Sammlung von Mikroorganismen und Zellkulturen), Artificial Seawater) [37], artificial urine spiked with 10^5^ CFU/mL of uropathogenic *E. coli* (test sample), and artificial urine without uropathogenic *E. coli* were added to the wells (in triplicates), mixed with the previously lyophilized cells of *P. leiognathi* in the CUBET attachment cartridge, and incubated at room temperature for 5 min. After incubation, the cartridge was placed in the area allotted within the CUBET platform. A high-resolution smartphone camera was placed on top of the platform, and, with the use of long exposure photography, a picture was taken and analyzed using ImageJ. 1.x software [46]. The same procedure was performed using a luminometer (CLARIOstar, BMG Labtech; Cary, NC, USA). The two results were compared using one-way ANOVA with a significance cutoff of *p* ≤ 0.0001.

## 4. Conclusions

This study demonstrated two non-competing approaches to diagnosing UTI: TuBETUr and CUBET. Both technologies relied on the same “blackout” phenomenon that resulted from exposure of a bioluminescence bacteria to an infected urine sample. TuBETUr relied on active *P. mandapamensis* for use in a laboratory environment, while CUBET was designed around the reconstitution of lyophilized *P. leiognathi* to perform the same diagnostic task in a point-of-care setting. Both assays provided an extremely rapid and affordable contrast to standard culture assays and nucleic acid amplification tests, and they were capable of meeting or exceeding the clinical metric of ≥ 10^5^ CFU/mL detection for a positive diagnosis. Most importantly, these exceptional diagnostic tools could significantly reduce the prevalence of antibiotic resistance through the rapid confirmation of infection, as UTIs rank among the most common infections requiring antibiotics.

## Figures and Tables

**Table 1:** Time of blackout for the four standard strains of uropathogens.

UROPATHOGEN	Time of Blackout (s)		Regression Equation (R^2^)	10^5^ cfu/mL (s) *
	10^2^ cfu/mL	10^8^ cfu/mL		
*Escherichia coli* ATCC 25922^TM^	1242	316	0.9859	831
*Staphylococcus aureus* ATCC 23235^TM^	1145	253	0.9953	663
*Proteus mirabilis* ATCC 35659^TM^	1431	238	0.9842	694
*Candida albicans* ATCC 14053^TM^	1978	392	0.9851	1246
**Cut-off time**	-	1257 s	-	-

* clinically accepted bacterial load for diagnosing urinary tract infection

## Supplementary Materials

Supplementary materials can be found at https://www.mdpi.com/1422-0067/21/14/5015/s1. Figure S1. Mean and standard deviation of blackout (in seconds) of the positive controls. Figure S2. Results of the determination for the mechanism of action. Acquisition and Lyophilization of P. leiognathi ATCC 33981TM. Figure S3. Lyophilization of Photobacterium leiognathi. Microtiter Plate Assay for Urinary Tract Infection. Figure S4. Urinary tract infection microtiter plate assay. Deoxygenation of Deionized Water and Artificial Urine. Figure S5. Removal of oxygen in artificial urine. Table S1. Phenotypic characteristics of selected photobacterium. Table S2. Genotypic characterization of the isolated bacteria (Macrogen, Seoul, South Korea). Table S3. Patients profiles with UTI detected by tube bioluminescence extinction technology urine in 30 normal and 30 positive UTI samples detected by urine culture.

## Abbreviations

UTI	Urinary tract infection
TuBETUr	Tube bioluminescence extinction technology urine
CUBET	Cellphone-based UTI bioluminescence extinction technology
CFU	Colony-forming unit
TYESA	Trypticase yeast extract seawater agar
ATCC	American Type Culture Collection
USTCMS	University of Santo Tomas Collection of Microbial Strains
LE	Leukocyte esterase
NT	Nitrite test
FISH	Fluorescence in situ hybridization
ATP	Adenosine triphosphate
RNA	Ribonucleic acid
rRNA	Ribosomal ribonucleic acid
BLAST	Basic Local Alignment Search Tool
Bp	Base pair
pH	Potential of hydrogen
AHL	Acyl homoserine lactone
AI2	Autoinducer-2
GI	Gastrointestinal
APW	Alkaline peptone water
SIM	Sulfur indole motility
TSB	Trypticase soy broth
TCBS	Thiosulfate citrate bile salt sucrose
RLU	Relative light unit
OD	Optical density
rpm	Rotation per min
DSMZ	Deutsche Sammlung von Mikroorganismen und Zellkulturen
