# Supplementary material for: An Intact Cell Bioluminescence-Based Assay for the Simple and Rapid Diagnosis of Urinary Tract Infection
Source: Int J Mol Sci. 2020 Jul 16;21(14):5015. doi: 10.3390/ijms21145015 (PMC7404122; doi:10.3390/ijms21145015)
Supplement: Supplementary file 1 [file ijms-21-05015-s001.pdf]

International Journal of
*Molecular Sciences*

*Supplementary Materials*

# An Intact Cell Bioluminescence-Based Assay for the Simple and Rapid Diagnosis of Urinary Tract Infection

**Sherwin Reyes [1,2,3,4], Nga Le [1], Mary Denneth Fuentes [3,4], Jonathan Upegui [1], Emre Dikici [1,2], David Broyles [1,2], Edward Quinto [4,†], Sylvia Daunert [1,2,5] and Sapna K. Deo [1,2,*]**

1 Department of Biochemistry and Molecular Biology, University of Miami Miller School of Medicine, Miami, Florida 33136, USA; sxr1145@miami.edu (S.R.); ntl21@miami.edu (N.L.); jupegui@miami.edu (J.U.); EDikici@med.miami.edu (E.D.); DBroyles@med.miami.edu (D.B.); SDaunert@med.miami.edu (S.D.); SDeo@med.miami.edu (S.K.D.)
2 The Dr. John T. McDonald Foundation Bionanotechnology Institute of University of Miami, Florida 33136, USA
3 FEU-Nicanor Reyes Medical Foundation, Institute of Medicine, West Fairview, Quezon City 1118, Philippines; marydenfuentes@gmail.com
4 University of Santo Tomas, The Graduate School, Espana, Manila 1015, Philippines; edwardquinto@gmail.com
5 Clinical and Translational Science Institute of University of Miami, Florida 33136, USA
† Deceased: 9 August 2017
* Correspondence: SDeo@med.miami.edu; Tel.: +1-(305)-243-4421

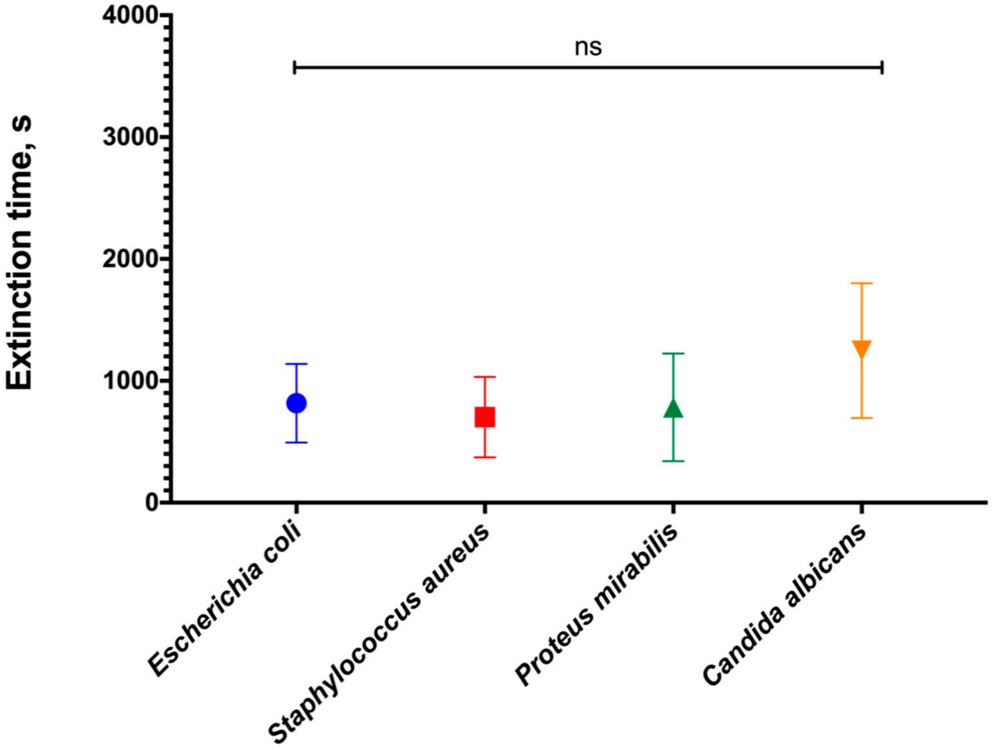


**Figure S1.** Mean and standard deviation of blackout (s) of the positive controls.

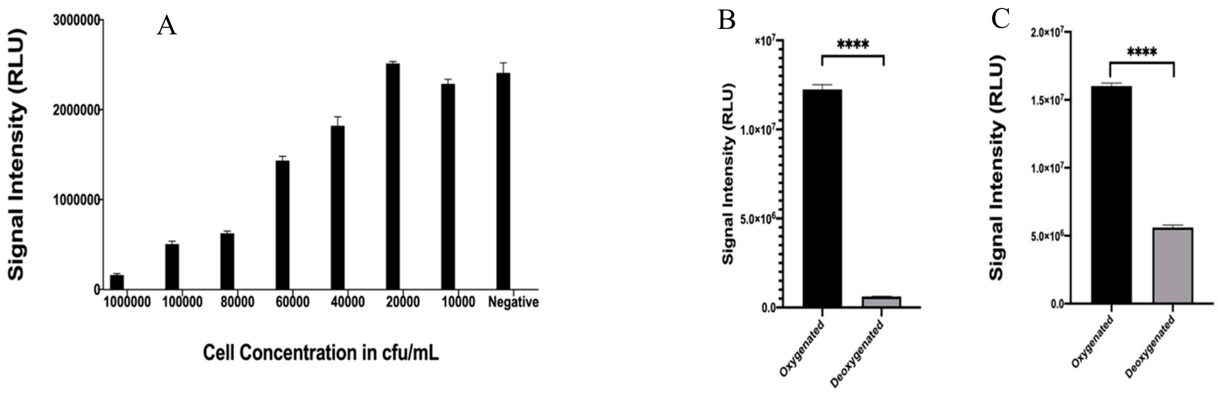


**Figure S2.** Results of the determination for the mechanism of action. (**A**) The blackout of lyophilized *P. leiognathi* on artificial urine spiked with NEB® 5-alpha cells, (**B**) Luminescence of oxygenated and deoxygenated ionized water, (**C**) Luminescence of oxygenated and deoxygenated artificial urine. Both B and C were subjected to deoxygenation using argon gas purging.

## Acquisition and Lyophilization of *P. leiognathi* ATCC 33981™

*P. leiognathi* ATCC 33981™ was procured from the repository of the American Type Culture Collection (ATCC). *P. leiognathi* was utilized to replace *P. mandapamensis* due to limited accessibility, as *P. leiognathi* and *P. mandapamensis* are phenotypically identical organisms [22,23,32,33]. Therefore, this strain performed the same role in the microtiter plate assay and point-of-care testing using the cellphone-based urinary tract infection bioluminescence extinction technology (CUBET) as *P. mandapamensis*. For lyophilization (microtiter plate assay), (Figure S3) 250 mL AB medium and 2 mL of an overnight culture of *P. leiognathi* cells were transferred to a 500 mL flask, and the culture was incubated at room temperature with shaking until the optical density ($OD_{600}$) reached 0.711. A 40 mL aliquot of the bacterial suspension was then transferred to a sterile 50 mL conical vial and centrifuged for 5 min at 3000 × *g* and 4 °C. The pellet was isolated by decanting the supernatant and was resuspended to homogeneity by vortexing in 40 mL of SF3 cryoprotectant (120 g/L lactose, 20 g/L soluble starch, and 10 g/L sodium chloride dissolved in deionized water and adjusted to pH 7). To 4 mL amber bottle, 200 µL of the mixture was transferred and lyophilized at -80 °C for 48 h. A similar procedure was done for the lyophilization of cells in the CUBET platform, only that 200 µL of the cell suspension was transferred into the wells of the CUBET attachment cartridge and placed at −80 °C for 2 h. The frozen cells were then lyophilized for 24–48 h. After freeze-drying, the cartridge was wrapped using clean plastic film and stored at room temperature until usage.

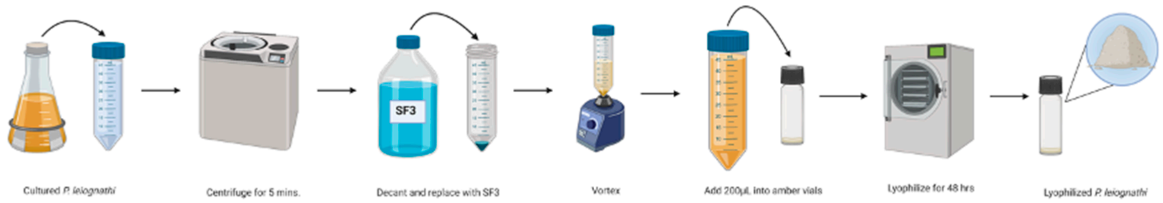


**Figure S3.** Lyophilization of *Photobacterium leiognathi*.

## Microtiter Plate Assay for Urinary Tract Infection

Overnight cultures of *E. coli* were prepared for assay by performing 10-fold serial dilutions from $10^6$ to $10^3$ CFU/mL using artificial urine (Figure S4). The optical density of *E. coli* was measured and computed using a standard curve in order to convert from optical density to colony-forming units. After dilution, the cells were incubated at 37 °C for 1 h. In a sterile, black 96-well flat bottom (chimney well) Cellstar®TC plate, 10 µL of the freshly reconstituted *P. leiognathi* cells and 190 µL of the

incubated *E. coli* cells were added per well in triplicate for each dilution. The plate was then mixed and read by a luminometer.

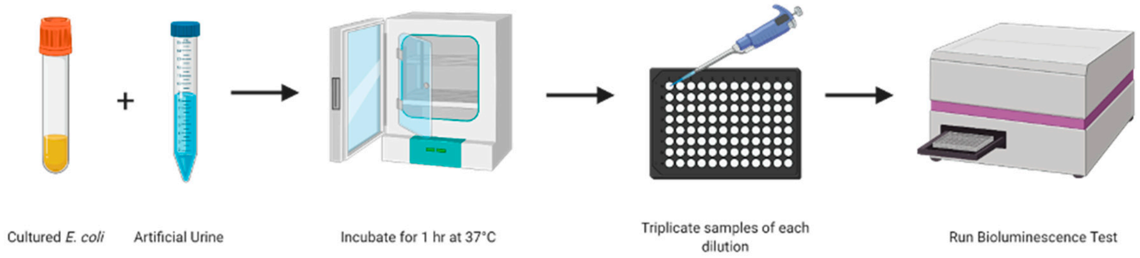


**Figure S4.** Urinary tract infection microtiter plate assay.

## Deoxygenation of Deionized Water and Artificial Urine

A deoxygenation apparatus was constructed by using a 250 mL round bottom flask with a stopcock and a septum on top (Figure S5). The flask was filled with either 100 mL of deionized water or commercially available artificial urine. The freezing process was performed in a dry ice/isopropanol bath at approximately −70 °C for 25–30 min or until the solution completely solidified. Following solidification, the flask was placed under vacuum for 15 min at −70 °C prior to thawing at room temperature while under an argon atmosphere. This was repeated three times to ensure complete deoxygenation.

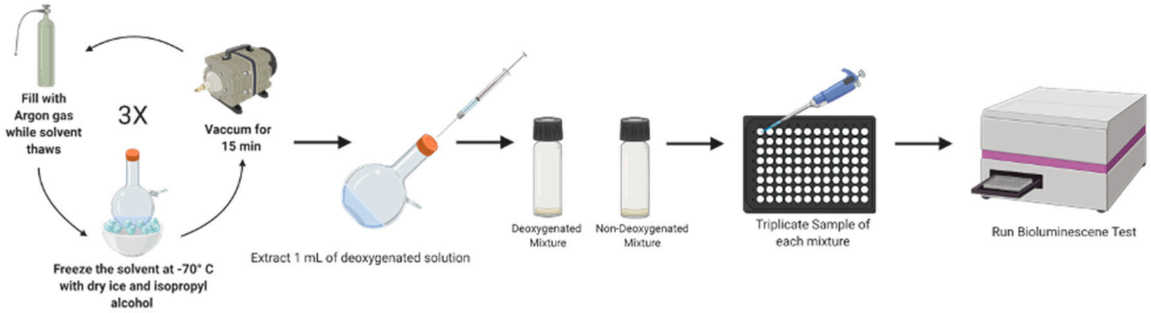


**Figure S5.** Removal of oxygen in artificial urine.

**Table S1.** Phenotypic characteristics of selected photobacterium.

| Test | Results |
|---|---|
| Gram | − |
| Indole | − |
| Methyl red | + |
| Voges–Proskauer | + |
| Citrate utilization | − |
| Gelatinase hydrolysis | − |
| Nitrate reduction | − |
| TCBS growth | − |
| Catalase | + |
| Glucose/lactose fermentation | + |
| Gas/hydrogen sulfide production | − |

**Table S2.** Genotypic characterization of the isolated bacteria (Macrogen, Seoul, South Korea).

| Accession Number | Identification | Score | | | Identities | | |
|---|---|---|---|---|---|---|---|
| | | Bit | Raw | E Value | Match | Total | Pct (%) |
| 130130-33_A01_BLB-13-01_518F | *Photobacterium mandapamensis* strain ATCC 33981 | 1718 | 930 | 0 | 947 | 954 | 99 |
| 130130-33_E01_BLB-13-01_518F | *Photobacterium mandapamensis* strain ATCC 33981 | 1705 | 923 | 0 | 940 | 947 | 99 |
| 130130-33_I01_BLB-13-01_518F | *Photobacterium sp.* FL3D2 | 1716 | 929 | 0 | 937 | 941 | 99 |
| 130130-33_M01_BLB-13-01_518F | *Photobacterium mandapamensis* strain ATCC 33981 | 1716 | 929 | 0 | 946 | 953 | 99 |

**Table S3.** Patients profiles with UTI detected by tube bioluminescence extinction technology urine in 30 normal and 30 positive UTI samples detected by urine culture.

| | ***n*** **= 59 (%)** | **Blackout Time (s)** |
|---|---|---|
| UTI NEGATIVE SAMPLES | 30 (100) | 1260 – 3660 |
| UTI POSITIVE SAMPLES | 29 (100) | < 1119 |
| Urine Colony Count of ≥ $10^5$ CFU/mL and the Pathogen Isolated | | |
| | ***n*** **= 29 (%)** | (lowest/highest) |
| GRAM NEGATIVE (Enterobacteriaceae) | | |
| *Lactose Fermenters* | 20 | |
| *Escherichia coli* | 14 (48.28) | <10 s/306 s |
| *Klebsiella pneumoniae* | 2 (7.14) | <10 s/258 s |
| *Enterobacter aerogenes* | 1 (3.44) | <10 s |
| *Enterobacter cloacae* | 2 (6.90) | <10 s/108 s |
| *Citrobacter freundii* | 1 (3.44) | <10 |
| *Non-Lactose Fermenters* | 1 | |
| *Proteus mirabilis* | 1 (6.90) | 417 s |
| *Non-Fermenters* | 2 | |
| *Acinetobacter baumannii* | 1 (3.44) | 32 s |
| *Burkholderia cepacia* | 1 (3.44) | <10 s |
| GRAM POSITIVE | 1 | |
| *Enterococcus faecalis* | 1 (3.44) | 140 s |
| *With antibiotic resistance* | 3 | |
| ***ESBL – Escherichia coli* | 1 (3.44) | <10 s |
| *ESBL – Klebsiella pneumoniae* | 1 (3.44) | <10 s |
| ****MR – Staphylococcus aureus* | 1 (3.44) | 53 s |
| *Mix isolates* | 2 | |
| *Citrobacter freundii*<br>*Escherichia coli* | 1 (3.44) | 27 s |
| *Escherichia coli*<br>*Acinetobacter baumannii* | 1 (3.44) | <10 s |

** ESBL (extended spectrum beta-lactamase) *** MR (methicillin-resistant).
